# Microbial oxidation of arsenite in a subarctic environment: diversity of arsenite oxidase genes and identification of a psychrotolerant arsenite oxidiser

**DOI:** 10.1186/1471-2180-10-205

**Published:** 2010-07-30

**Authors:** Thomas H Osborne, Heather E Jamieson, Karen A Hudson-Edwards, D Kirk Nordstrom, Stephen R Walker, Seamus A Ward, Joanne M Santini

**Affiliations:** 1 Institute of Structural and Molecular Biology, UCL, Darwin Building, Gower Street, London WC1E 6BT, UK; 2 Department of Geological Sciences and Geological Engineering, Queen's University, Kingston, Ontario K7L 3N6, Canada; 3 Department of Earth and Planetary Sciences, Birkbeck, University of London, Malet Street, London WC1E 7HX, UK; 4 US Geological Survey Boulder, Colorado 80303, USA; 5 Research Department of Genetics, Evolution and Environment, UCL, Darwin Building, Gower Street, London WC1E 6BT, UK

## Abstract

**Background:**

Arsenic is toxic to most living cells. The two soluble inorganic forms of arsenic are arsenite (+3) and arsenate (+5), with arsenite the more toxic. Prokaryotic metabolism of arsenic has been reported in both thermal and moderate environments and has been shown to be involved in the redox cycling of arsenic. No arsenic metabolism (either dissimilatory arsenate reduction or arsenite oxidation) has ever been reported in cold environments (i.e. < 10°C).

**Results:**

Our study site is located 512 kilometres south of the Arctic Circle in the Northwest Territories, Canada in an inactive gold mine which contains mine waste water in excess of 50 mM arsenic. Several thousand tonnes of arsenic trioxide dust are stored in underground chambers and microbial biofilms grow on the chamber walls below seepage points rich in arsenite-containing solutions. We compared the arsenite oxidisers in two subsamples (which differed in arsenite concentration) collected from one biofilm. 'Species' (sequence) richness did not differ between subsamples, but the relative importance of the three identifiable clades did. An arsenite-oxidising bacterium (designated GM1) was isolated, and was shown to oxidise arsenite in the early exponential growth phase and to grow at a broad range of temperatures (4-25°C). Its arsenite oxidase was constitutively expressed and functioned over a broad temperature range.

**Conclusions:**

The diversity of arsenite oxidisers does not significantly differ from two subsamples of a microbial biofilm that vary in arsenite concentrations. GM1 is the first psychrotolerant arsenite oxidiser to be isolated with the ability to grow below 10°C. This ability to grow at low temperatures could be harnessed for arsenic bioremediation in moderate to cold climates.

## Background

Arsenic's toxic and medicinal properties have been appreciated for more than two millennia [1]. Its two soluble inorganic forms, arsenite (+3) and arsenate (+5), entering drinking water from natural sources, have caused poisoning in Taiwan, Chile, Argentina, Bangladesh and West Bengal, and most recently arsenicosis (arsenic poisoning) has been detected in people from Cambodia, Vietnam, Nepal, China, Inner Mongolia, Bolivia and Mexico [2,3]. In addition, arsenic contamination due to anthropogenic activity (e.g. mining) is increasing in importance in parts of the USA, Canada, Australia, Argentina and Mexico [4]. Although arsenic is toxic to most organisms, some prokaryotes have evolved mechanisms to gain energy by either oxidising or reducing it [5,6].

Prokaryotic arsenic metabolism has been detected in hydrothermal and temperate environments and has been shown to be involved in the redox cycling of arsenic [7-10]. The arsenite-oxidising bacteria isolated so far are phylogenetically diverse. The oxidation of arsenite may yield useable energy or may merely form part of a detoxification process [6]. To date, all aerobic arsenite oxidation involves the arsenite oxidase that contains two heterologous subunits: AroA (also known as AoxB) and AroB (also known as AoxA) [6]. AroA is the large catalytic subunit that contains the molybdenum cofactor and a 3Fe-4S cluster and AroB contains a Rieske 2Fe-2S cluster [6].

Although arsenic metabolism has been detected in both moderate and high-temperature environments, and mesophilic and thermophilic arsenite oxidisers have been isolated, no arsenic metabolism (either dissimilatory arsenate reduction or arsenite oxidation) has ever been detected in cold environments (i.e. < 10°C). One such environment with high concentrations of arsenic is the Giant Mine, one of Canada's oldest and largest gold mines. It is located a few kilometres north of Yellowknife, Northwest Territories, 62° north of the equator and 512 kilometres south of the Arctic Circle. Gold was produced from 1948 to 1999 by roasting arsenopyrite (FeAsS)-bearing ore. The mine now contains approximately 300,000 tonnes of arsenic trioxide, stored in underground chambers [11]. Temperatures in the underground stopes range from 4°C to 10°C [11].

Here we report the detection, isolation and characterisation of an aerobic psychrotolerant arsenite-oxidising bacterium from a subterranean biofilm in the Giant Mine. Unlike other characterised arsenite oxidisers, this organism is capable of growing below 10°C and is the first heterotrophic organism to oxidise arsenite in the early exponential phase of growth. We also compare the diversity of arsenite oxidisers in two subsamples of the biofilm that vary in arsenite concentrations.

## Results and Discussion

**Figure 1:**
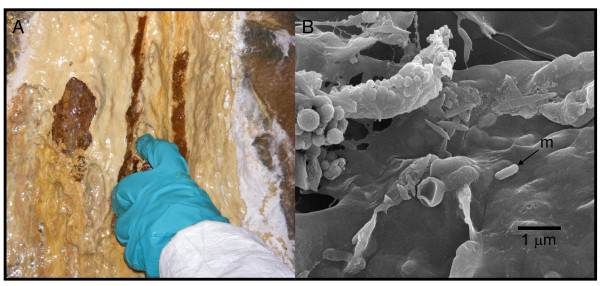
**Microbial biofilm sampled from Giant Mine, Yellowknife, NWT, Canada**. (A) Microbial biofilm. The mineral yukonite, a Ca-Fe arsenate is shown by the reddish-brown colouration. (B) Scanning electron micrograph of biofilm showing extracellular polymeric substance (EPS) which appear as threads and microbes (m).

The Giant Mine has a long history of arsenic contamination and dissolution of stored arsenic trioxide by infiltrating groundwaters has increased arsenic concentrations at this site from a few to 50 mM. Biofilms have formed at many places where water seeps into the underground excavations [11]. One such biofilm (Figure 1a) was located growing in an abandoned stope below seepage from a diamond drill hole approximately 152 m below the arsenic trioxide chambers (230 m below land surface) (temperature at each time of sampling was *ca*. 4°C). Water taken from the top of the biofilm in 2006 contained 14.01 mM total soluble arsenic and 2.56 mM arsenite. Samples taken in 2007 from the top and bottom of the biofilm contained 9.57 mM total soluble arsenic and 9.22 mM arsenite (top) and 9.16 mM total soluble arsenic and 6.01 mM arsenite (bottom). The concentration of arsenite in the 2006 sample was substantially lower than that of the equivalent top sample from 2007. The reason for this was probably microbial arsenite oxidation during storage as the liquid was not extracted from the 2006 sample until 18 days after collection whereas the liquid was extracted immediately from the 2007 samples. SEM examination of the biofilm revealed the presence of threadlike extracellular polymeric substances and distinct microorganisms (Figure 1b).

**Figure 2:**
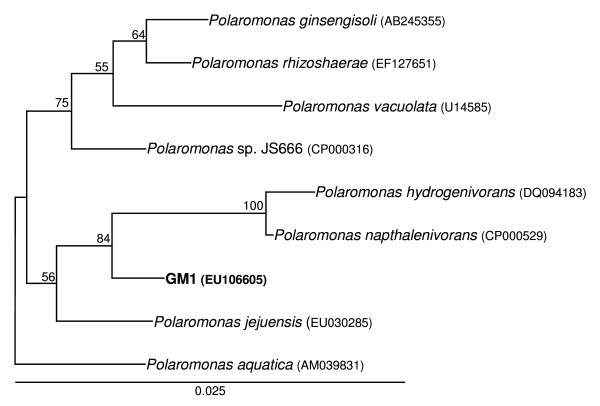
**16S rRNA phylogenetic tree of arsenite-oxidising strain GM1 and published *Polaromonas *species**. GenBank accession numbers are in parentheses. Significant bootstrap values (per 100 trials) are shown. The tree is rooted with the 16S rRNA gene sequence of *Alcaligenes faecalis *(AY027506) (not shown).

The arsenite-oxidising bacterium, designated GM1 was isolated and found to be a Gram-negative, rod-shaped, motile, heterotroph. Phylogenetic analysis of its full 16S rRNA gene sequence (Figure 2) showed it to be a member of the Betaproteobacteria related to *Polaromonas *species. GM1 is closely related (98% sequence identity) to *Polaromonas *sp. JS666, a *cis*-dichloroethene-degrading bacterium isolated from granular activated carbon from Dortmund, Germany [12], and *Polaromonas napthalenivorans *CJ2 a naphthalene-degrading bacterium isolated from a coal-tar contaminated aquifer in New York state, USA [13]. Using the CLASSIFIER tool of the Ribosomal Database Project we classed GM1 as a *Polaromonas *species [14] and the first capable of oxidising arsenite. Unlike its phylogenetic relatives GM1 was unable to grow with either *cis*-dichloroethene or naphthalene as sole carbon source (data not shown).

**Figure 3:**
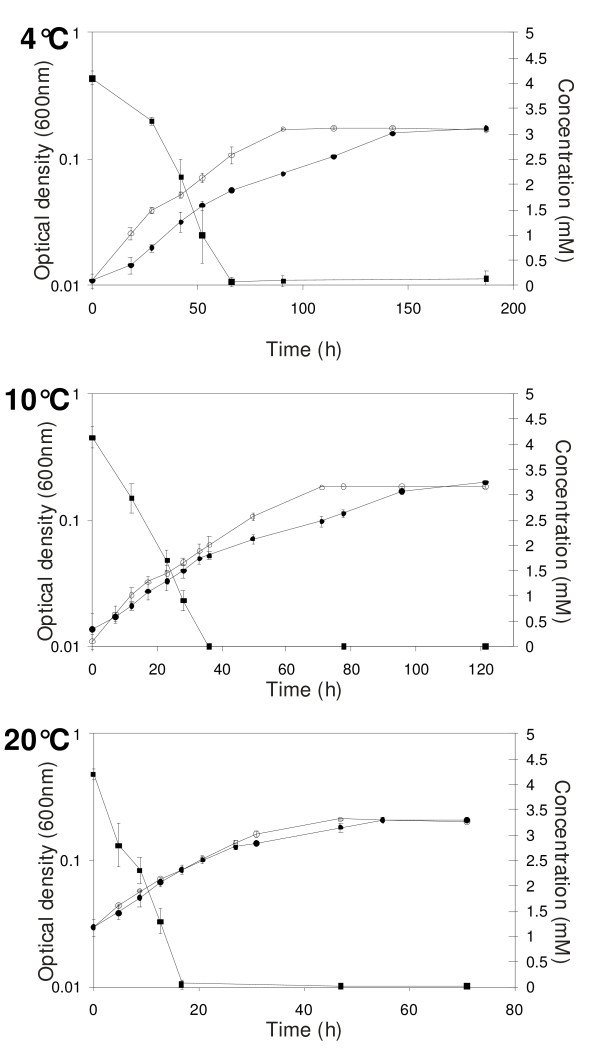
**Growth curves of GM1 grown at 4°C, 10°C and 20°C in the Minimal Salts Medium (MSM) with 0.04% (w/v) yeast extract**. With 4 mM arsenite, closed circle; without arsenite, open circle; arsenite concentration, closed square. Error bars are the standard deviation of multiple experiments.

Growth of GM1 was tested at 4°C, 10°C and 20°C in a minimal salts medium (MSM) with 0.04% (w/v) yeast extract in the presence and absence of 4 mM arsenite as described previously [15] (Note: GM1 was unable to grow chemolithoautotrophically with arsenite). Under all conditions arsenite was oxidised to arsenate and oxidation occurred in the early exponential phase of growth (Figure 3). The generation time of GM1 was shorter in the absence of arsenite, and decreased with increasing temperature (without arsenite at 4°C, 10°C and 20°C: 19 h, 16.5 h and 7 h, respectively; with arsenite at 4°C, 10°C and 20°C: 21.5 h, 17.7 h and 8.5 h, respectively). GM1 did not grow above 25°C. To date, only one arsenite oxidiser has been demonstrated to grow below 20°C [16]. This organism, a chemolithoautotrophic arsenite oxidiser designated M14, is a member of the Alphaproteobacteria related to *Sinorhizobium *species. M14's temperature range was between 10°C and 37°C with an optimum of 22°C [16]. GM1 is the first reported arsenite oxidiser capable of growth below 10°C.

The arsenite-oxidising ability of GM1 was further confirmed by testing for arsenite oxidase (Aro) activity in cells grown in the MSM with 4 mM arsenite and 0.04% (w/v) yeast extract. Aro activity was measured at room temperature (i.e. 24°C) in its optimal buffer, 50 mM 2-(N-Morpholino)ethanesulfonic acid (MES) (pH 5.5) (data not shown). Aro activity was higher when GM1 was grown at 10°C (0.334 U/mg) compared with growth at 4°C (0.247 U/mg) and 20°C (0.219 U/mg) which were comparable. In growth experiments although all the arsenite is oxidised to arsenate in the early exponential growth phase the highest Aro activity was observed in the stationary phase of growth (i.e. 0.334 U/mg compared with 0.236 U/mg at early exponential phase).

**Figure 4:**
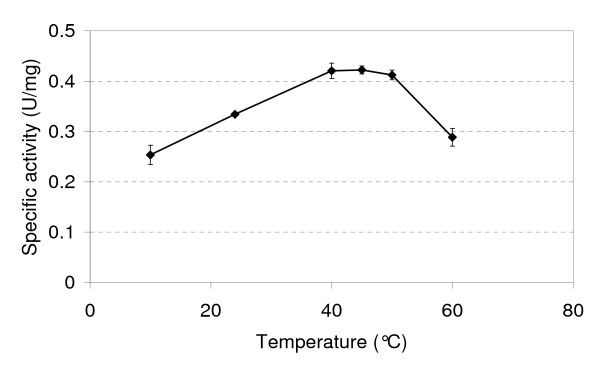
**Specific activity of GM1 arsenite oxidase as a function of temperature**. Error bars are the standard deviation of multiple assays.

In most cases, arsenite is required in the growth medium for arsenite oxidase gene expression [6]. There are two exceptions, *Thiomonas *sp. str. 3As and *Agrobacterium tumefaciens *str. 5A, where the arsenite oxidase is expressed when the organisms are grown in the absence of arsenite but in the latter the expression does not occur until stationary phase [17,18]. In GM1 arsenite oxidase expression is also constitutive when grown in the absence of arsenite [i.e. in the MSM with 0.04% (w/v) yeast extract] with 0.367 U/mg observed in late exponential phase and activity also detected in early exponential phase (0.13 U/mg). Taken together this information suggests that there are at least two modes of regulating the expression of the *aro *genes in GM1, possibly a two-component signal transduction system and quorum sensing. Because of the broad temperature range for growth of GM1, arsenite oxidase activity was determined at a variety of temperatures (Figure 4). Activity occurred over a broad temperature range reaching a maximum at temperatures well above the optimum for growth (i.e. between 40-50°C).

The partial *aroA *gene sequence of GM1 was found to be identical to that of the partial *aroA *of the putative arsenite oxidiser *Limnobacter *sp. 83, another member of the Betaproteobacteria [8] but in a different family. No homologues of *aroA *were found in the genome sequences of GM1's closest relatives, *Polaromonas naphthalenivorans *CJ2 and *Polaromonas *sp. JS666; GM1 is thus clearly distinct from the other *Polaromonas *spp.

**Figure 5:**
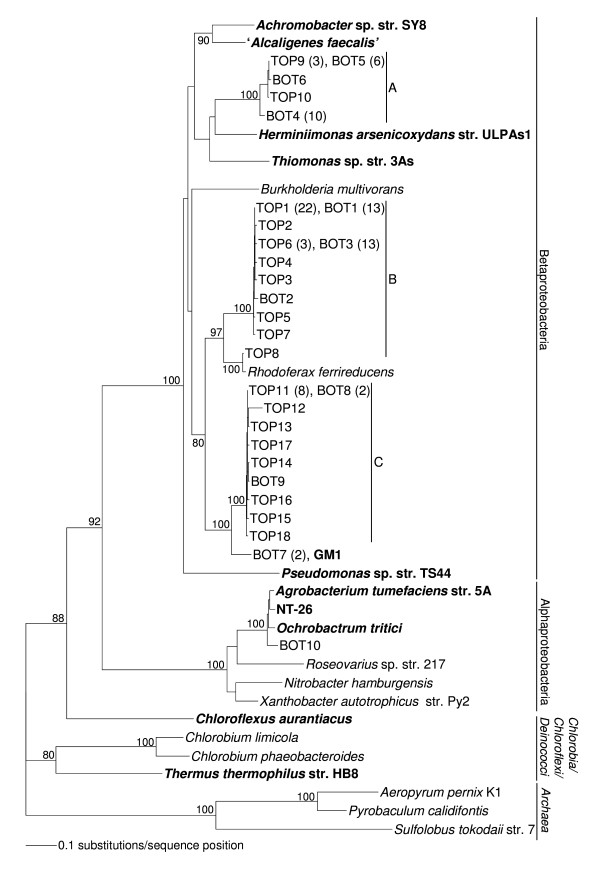
**Phylogenetic tree of AroA-like sequences from an arsenic-contaminated biofilm**. Phylogenetic analysis of 50 AroA-like sequences from both the top (TOP) and bottom (BOT) of the biofilm. Published AroA sequences are in bold, organisms that contain AroA homologues and the AroA from the arsenite-oxidising bacterium GM1 are also shown. Numbers in parentheses indicate the number of identical sequences represented by each branch. Significant bootstrap values (per 100 trials) of major branch points are shown. Closely related groups of sequences have been designated clades A, B and C. Putative AroA sequences from the Archaea were used to root the tree.

To compare the arsenite oxidisers in the top (9.22 mM arsenite) and bottom (6.01 mM arsenite) subsamples from the 2007 biofilm, two *aroA *gene libraries were constructed using a recently developed method [7]. The use of *aroA*-specific primers has been shown to be a useful approach for detecting and identifying arsenite oxidisers in environmental samples [7-10,19]. Phylogenetic analysis of 100 AroA-like sequences (Figure 5), from 50 top (designated TOP) and 50 bottom (designated BOT) clones, revealed the diversity of arsenite-oxidising bacteria in the two subsamples. The corresponding protein sequences were compared with known and putative AroA sequences and with the sequence obtained from GM1. Eighteen different AroA-like sequences were obtained from the TOP library and ten from BOT; only four were present in both. All but one of the sequences clustered within the Betaproteobacteria; the exception, BOT10, clustered within the *Agrobacterium*/*Rhizobium *branch of the Alphaproteobacteria. The TOP8 sequence is closely related (98.7% sequence identity) to the AroA homologue in *Rhodoferax ferrireducens*. Apart from BOT10 the AroA-like sequences clustered into three distinct clades (A, B and C), none of which is close to any AroA sequences from known arsenite oxidisers. The BOT7 sequence (clade C) was identical to the AroA sequence of GM1, so the other sequences in clade C may also come from *Polaromonas *species. The affinities of the organisms whose AroA sequences lie in clades A and B are not known.

**Figure 6:**
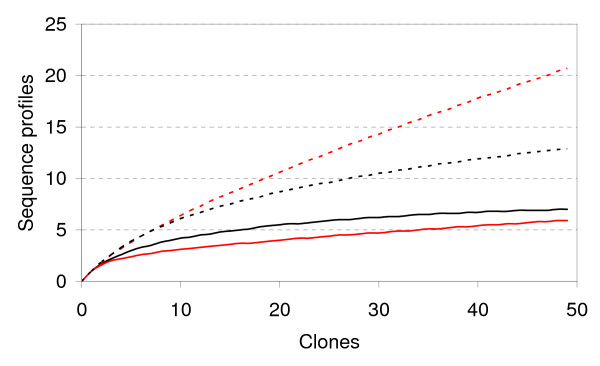
**Rarerefaction curves for DNA sequences from *aroA*-like gene libraries TOP (red) and BOT (black)**. Dashed lines are for different sequence profiles. Solid lines are for OTUs based on > 99% sequence identity.

Rarefaction curves (Figure 6) of different DNA sequence profiles suggest that the TOP library has higher sequence richness (i.e. more distinct sequences) than the BOT library. Curve saturation was not observed for either library, suggesting that not all of the *aroA*-like genes present had been detected. A separate rarefaction analysis was performed on the operational taxonomic units (OTUs), where sequences were clustered with BLASTclust based on a 99% identity threshold. Both OTU curves come close to saturation, approaching similar richness asymptotes; *aroA*-like OTU richness is similar in TOP and BOT (BOT appears to be slightly more diverse, but the 95% confidence intervals showed that there was no significant difference). While 50 clones may not have yielded the full sequence richness of either library, continued sampling would have been unlikely to reveal significant numbers of additional OTUs.

With almost all sequences represented by only a single clone (Figure 5) sequence diversity (evenness) is inevitably high in both subsamples. Simpson's index [20] does not differ between them (TOP: D = 0.78; BOT: D = 0.82). The two subsamples do, however, differ in composition. They are dominated by clones from different clades: TOP by clades B and C; BOT by A and B (Table 1: χ^2 ^= 16.17, 2 d.f. *P *< .001). The difference reflects the numbers of clones from the three clades, rather than the distribution of the sequences.

**Table 1:** The number of clones from TOP and BOT that clustered within clades A, B and C

Clade	TOP	BOT	Total
A (%)	4 (19%)	17 (81%)	**21**
B (%)	30 (53%)	27 (47%)	**57**
C (%)	15 (83%)	3 (17%)	**18**

## Conclusions

In this report we provide the first evidence for bacterial arsenite oxidation below 10°C. The sample site, the Giant Mine, is an extreme environment with arsenic concentrations in excess of 50 mM in the underground waters [21]. In this study we have compared the diversity of arsenite oxidisers in two different subsamples and found that although the composition of arsenite-oxidising communities differs, the diversity does not. The isolated arsenite-oxidising bacterium GM1 was able to grow at low temperatures (< 10°C); its arsenite oxidase was constitutively expressed and displayed broad thermolability.

## Methods

### Sample collection and analyses

Samples were collected from Giant Mine, north of Yellowknife, Northwest Territories, Canada. The microbial biofilm was located growing on a wall in an abandoned stope below the arsenic trioxide storage chambers where liquid was seeping from a diamond drill hole. The first sampling of the biofilm was done in July 2006 and involved collecting some of the biofilm itself, coexisting seepage water, and mineral precipitates from near the top of the biofilm. The biofilm was re-sampled in May 2007 using the same sampling method as in 2006 but this time two samples were collected: one at the top near the seepage point and another near the bottom. All samples were kept at 4°C at all times until microbial or chemical analyses could be performed.

The 2006 biofilm sample was used for mineral characterisation. Mineral precipitates were characterised using beamline X26A at the National Synchrotron Light Source. MicroXANES (at the arsenic K edge) and microXRD followed methods similar to those described previously [22]. The XANES spectra collected on thin layers on sample powder provided clear indication of the presence of both arsenite and arsenate, and a linear combination fit, using scorodite (As^V^) and schneiderhohnite (As^III^) as model compounds, estimated the relative proportions at 57% arsenate and 43% arsenite. Synchotron-based microXRD of the biofilm showed clear evidence of microcrystalline yukonite, a Ca-Fe arsenate [Ca_7_Fe(AsO_4_)_9_O_10_·24.3H_2_O] [22] (see reddish-brown colouration in Figure 1a), gypsum and an arsenite mineral [either claudetite (As_2_O_3_) or manganarsite (Mn_3_As_2_O_4_(OH)_4_)].

### Arsenic analyses

In 2006 the liquid from the biofilm was extracted 18 days after collection whereas in 2007 the liquid was extracted immediately after collection. The liquid was extracted using a syringe with a 0.22-μm filter. Concentrations of total arsenic and arsenite were determined by hydride generation atomic-absorption spectrometry (HG-AAS) using a Perkin Elmer - Analyst 300.

Cultures were analysed for total arsenic and arsenite using a JY Ultima 2C ICP-OES using the methods described previously [23-25].

### Scanning electron microscopy

Samples from the top and bottom of the 2007 microbial biofilm were examined using a Jeol JSM-6480LV high-performance, variable pressure analytical scanning electron microscope (SEM) operating in low-vacuum mode using 7-11 kV accelerating voltage and a spot size of 29 nm. Prior to examination, samples were mounted on 12.5-mm pin stubs with sticky carbon discs, freeze-dried in liquid nitrogen using a MODULO 4 k instrument for 30 minutes, and gold coated using a Polaron E5000 instrument.

### Enrichment and isolation

In 2006 samples of the microbial biofilm (0.5 g) were inoculated into the MSM [15] containing 4 mM arsenite and incubated at 4°C, 10°C and 20°C. The enrichments were incubated until all the arsenite was oxidised. The biofilm enrichments took two days to oxidise the 4 mM arsenite irrespective of temperature (data not shown). The enrichments were subcultured three times in the MSM containing 4 mM arsenite before they were serially diluted and plated onto MSM containing 4 mM arsenite and 1.5% (w/v) purified agar (Oxoid). Individual colonies were purified and tested for both chemolithoautotrophic [containing 0.05% (w/v) NaHCO_3 _as carbon source] and heterotrophic (containing 0.04% (w/v) yeast extract) growth with arsenite [15].

### Growth of GM1

Growth experiments of GM1 were conducted in MSM containing 0.04% (w/v) yeast extract in the presence and absence of 4 mM arsenite at 4°C, 10°C and 20°C with shaking at 130 rpm in batch cultures. Experiments were commenced with a 5% (v/v) inoculum of late exponential phase cells grown in the same medium at the same temperature. At regular time intervals samples were taken to measure optical density and pH, and for arsenic analyses. Samples for arsenic analyses were centrifuged in a bench-top centrifuge and the supernatant stored at -20°C until required. All growth experiments were performed on at least two separate occasions with two to three replicates.

### Arsenite oxidase assays

GM1 cultures were harvested and crude cell extracts produced by passing them through a French pressure cell at 14 kPSI and arsenite oxidase activity determined by measuring the reduction of the artificial electron acceptor 2,6-dichlorophenolindophenol [15]. All assays were performed in the optimum buffer for the enzyme, 50 mM MES buffer (pH 5.5). Reactions were incubated at the specific temperature with a Cary Dual Cell Peltier for 5 mins prior to the addition of arsenite.

### 16S rRNA gene sequence determination and phylogenetic analyses

Genomic DNA was extracted using the Wizard^® ^Genomic DNA purification kit (Promega). 16S rDNA was amplified by PCR using the 27f and 1525r primers described previously [26], with Phusion high fidelity DNA polymerase (New England Biolabs) under the following conditions: 98°C for 30 s, followed by 40 cycles of 98°C for 30 s, 55°C for 30 s and 72°C for 90 s with a final extension at 72°C for 10 min. Both strands of the PCR product were sequenced at the Wolfson Institute for Biomedical Research (WIBR) (UCL) using the primers 27f, 342r, 357f, 518r, 530f, 1100r, 1114f, 1392r, 1406f, 1492r and 1525r [26]. [GM1 16S rRNA gene sequence GenBank accession number: EU106605].

### Amplification of *aroA*, library construction and sequencing

Genomic DNA was extracted from GM1 using the Wizard^® ^Genomic DNA purification kit (Promega) and from the top and bottom biofilm samples using the PowerSoil DNA isolation kit (MoBio Laboratories). The degenerate oligonucleotides used to amplify a portion of the *aroA *gene were primer set #2 as described previously [7] using Phusion high fidelity DNA polymerase (New England Biolabs). The *aroA *PCR products from GM1 and the two biofilm samples were cloned into pBluescript II KS+ (Stratagene). Both strands of the cloned GM1 *aroA *gene and 50 individual *aroA *clones from each library were sequenced using the T7 and T3 promoter primers at the WIBR (UCL). Database searches were performed using BLASTP [27]. [GM1 partial *aroA *sequence GenBank accession number: EU106602. The TOP and BOT *aroA *library sequences GenBank accession numbers: FJ151018-FJ151051].

### Phylogenetic analysis

Sequences were aligned with CLUSTALX 2.0 [28] using default settings and were manually edited. Phylogenetic analyses were performed with PHYLIP 3.67 [29] and trees constructed and edited with TREEVIEW [30]. Nucleotide and protein distance analyses were performed with the F84 and Jones-Taylor-Thornton computations, respectively and the trees constructed using the neighbour-joining method using a boostrap value of 100.

Accession numbers of reference sequences used in AroA phylogenetic analysis are given in parentheses following the organism name: *Achromobacter *sp. str. SY8 (ABP63660), *Aeropynum pernix *(NP_148692), *Agrobacterium tumefaciens *str. 5A (ABB51928), '*Alcaligenes faecalis*' (AAQ19838), *Burkholderia multivorans *(YP_001585661), *Chlorobium limicola *(ZP_00512468), *Chlorobium phaeobacteroides *(ZP_00530522), *Chloroflexus aurantiacus *(YP_001634827), *Herminiimonas arsenicoxydans *(YP_001098817), *Nitrobacter hamburgensis *(YP_571843), NT-26 (AAR05656), *Ochrobacterum tritici *(ACK38267), *Pseudomonas *sp. str. TS44 (ACB05943)*, Pyrobaculum calidifontis *(YP_001056256), *Rhodoferax ferrireducens *(YP_524325), *Roseovarius *sp. 217 (ZP_01034989), *Thermus thermophilus *str. HB8 (YP_145366), *Thiomonas *sp. 3As (CAM58792), *Sulfolobus tokodaii *str. 7 (NP_378391) and *Xanthobacter autotrophicus *Py2 (YP_001418831).

### Rarefaction curves and Chi-squared

Rarefaction calculations were performed to compare the DNA sequence diversity of the TOP and BOT libraries, and to assess whether full coverage of sequence diversity was obtained. This was performed with the program ANALYTICAL RAREFACTION 1.3 http://www.uga.edu/~strata/software/index.html which uses the rarefaction calculations given by Hulbert [31] and Tipper [32]. Sequences were clustered with BLASTclust http://toolkit.tuebingen.mpg.de/blastclust# based on a 99% identity threshold over 100% of the sequence length to create operating taxonomic units.

## Authors' contributions

THO performed the majority of the experiments (clone libraries, 16S rRNA gene sequencing, phylogenetic analyses, GM1 growth experiments and enzyme assays). HEJ collected the samples from Giant Mine and oversaw the mineral characterisation. KAH-E did the arsenic analyses for the growth experiments. SRW performed the mineral characterisation of the biofilm. DKN oversaw the chemical analyses of the biofilm samples. SAW advised on the statistical analyses and edited the manuscript. JMS isolated GM1 and the DNA from the biofilm, conceived and coordinated the study. All authors read and approved the final version of the manuscript.
